# Subjective thirst moderates changes in speed of responding associated with water consumption

**DOI:** 10.3389/fnhum.2013.00363

**Published:** 2013-07-16

**Authors:** Caroline J. Edmonds, Rosanna Crombie, Mark R. Gardner

**Affiliations:** ^1^School of Psychology, University of East LondonLondon, UK; ^2^Department of Psychology, University of WestminsterLondon, UK

**Keywords:** cognition, water, performance, mood, thirst

## Abstract

Participants (*N* = 34) undertook a CANTAB battery on two separate occasions after fasting and abstaining from fluid intake since the previous evening. On one occasion they were offered 500 ml water shortly before testing, and on the other occasion no water was consumed prior to testing. Reaction times, as measured by Simple Reaction Time (SRT), were faster on the occasion on which they consumed water. Furthermore, subjective thirst was found to moderate the effect of water consumption on speed of responding. Response latencies in the SRT task were greater under the “no water” condition than under the “water” condition, but only for those participants with relatively high subjective thirst after abstaining from fluid intake overnight. For those participants with relatively low subjective thirst, latencies were unaffected by water consumption, and were similarly fast as those recorded for thirsty participants who had consumed water. These results reveal the novel finding that subjective thirst moderates the positive effect of fluid consumption on speed of responding. The results also showed evidence that practice also affected task performance. These results imply that, for speed of responding at least, the positive effects of water supplementation may result from an attenuation of the central processing resources consumed by the subjective sensation of thirst that otherwise impair the execution of speeded cognitive processes.

## Introduction

Does having a drink help you think? Anecdotal evidence suggests that water consumption can help cognitive performance and recent research has supported this folk wisdom. This paper reports a study that examines the effect of water supplementation on cognitive performance and mood in adults, and examines whether there is a moderating effect of thirst. While the literature on the effects of water supplementation on cognition is rapidly growing, it is currently not vast. Therefore, this introduction will review the complementary literature on the negative effects of dehydration on cognition and mood, and the effect of additional water on cognition and mood in both adults and children.

There is some, if conflicting, evidence to suggest that dehydration negatively affects cognitive performance in adults (Benton, 2011). For example, dehydration to more than 1% loss of body weight resulted in poorer performance on a visual vigilance task and slower reaction times on a working memory task (Ganio et al., 2011). Some have found evidence suggestive of a dose response effect, with performance decreasing with increasing levels of dehydration (Sharma et al., 1986; Gopinathan et al., 1988). However, others have found that dehydration due to water deprivation does not affect cognitive performance (Szinnai et al., 2005), perhaps because dehydration caused by water deprivation takes some time to develop and participants may adapt during this period. Subjective ratings of cognitive performance and mood have been shown to be affected by dehydration. For example, reported alertness, and concentration worsen in dehydrated individuals (Shirrefs et al., 2004; Szinnai et al., 2005). Furthermore, adults judged that they were more fatigued and anxious when dehydrated (Ganio et al., 2011).

There are fewer studies assessing the effects of dehydration on cognition and mood in children; this is in part because it is not viewed as morally acceptable to deliberately dehydrate children. Thus, those studies that have been conducted have focused on children who happen to be dehydrated because they live in a hot climate. Studies conducted in Israel (Bar-David et al., 2005) and Italy (Fadda et al., 2012) have reported that a large proportion of children arrive at school in a dehydrated state (63 and 84%, respectively), and that there is a relationship between hydration status and memory, with children who are dehydrated having shorter digit spans than those who are better hydrated. Recent evidence suggests that around two-thirds of children in more temperate climes, including the UK, France, and the USA, may arrive at school dehydrated (as measured by urine osmolality over 800 mOsmol/kg of water) (Bonnet et al., 2012; Friedlander, 2012; Stookey et al., 2012), but these studies did not examine associations with cognitive performance.

Given that dehydration negatively affects cognitive performance, one might expect that supplementing with fluid would improve performance. In contrast to the few studies examining the effect of dehydration on cognitive performance in children, the majority of research on the effects of water supplementation on cognition has examined children. Offering children additional drinking water has a positive effect on their cognitive performance, particularly on tasks that require speeded processing or memory. For example, children performed better on tests assessing visual memory (measure by a consecutive spot the difference task) if they had consumed water (250 ml offered) (Edmonds and Burford, 2009). Similarly, children's verbal recall of objects was better on the occasion on which they consumed additional water (300 ml offered) (Benton and Burgess, 2009). Performance on tasks requiring visual attention and processing speed (letter cancellation) seem particularly sensitive to water supplementation (Edmonds and Burford, 2009; Edmonds and Jeffes, 2009; Booth et al., 2012). Reaction time has also been shown to be sensitive to water supplementation in children (Booth et al., 2012). One constant across the letter cancellation and reaction time tasks is that they both require speeded processing and fast responding. Thus, the current study includes a series of measures that assess these cognitive processes.

The cognitive performance of adults has also been shown to be improved by water supplementation. For example, performance on a rapid visual information processing task was improved by water consumption in a dose-dependent manner (120 or 330 ml offered), but only in those individuals who rated themselves as thirsty before drinking the water; if participants had low thirst initially, consuming water resulted in poorer performance (Rogers et al., 2001). Speeded processing has been shown to be improved by water consumption (200 ml offered), at both 20 and 40 min post consumption, while digit span and reaction time was not found to be affected by additional water (Edmonds et al., 2013). However, a third study found no relation between supplementation (120 ml or 300 ml offered) and performance on a range of cognitive tests, even when participants were grouped by initial thirst level (Neave et al., 2001). Water supplementation has not been found to impact on subjective measures of mood (Edmonds et al., 2013).

The present study investigated the effect of water supplementation on cognitive performance and mood in adults. We also considered whether subjective thirst moderates the relation between water supplementation and cognitive performance and mood. Given the range of cognitive processes shown to be affected by dehydration and water supplementation, a battery of tasks was administered via CANTAB. We controlled for baseline hydration status by having participants fast overnight before attending their test sessions, with no fluids being consumed since the previous evening. We expected water supplementation to result in improved performance on some of the cognitive test battery, and that thirst may mediate the effect of water.

## Methods

### Participants

Thirty-seven participants (25 female) were recruited. One did not return for the second testing session and so their results were discarded. The mean age was 29 years (range 20–53 years; *SD* = 8.3 years).

### Measures

#### Thirst scale

Our thirst scale asked, “how thirsty are you?” and participants marked a line to indicate their response. The line was labeled, “not hirsty at all” and “very thirsty” at opposite ends. A high score indicated greater subjective thirst.

#### Mood scale

The Visual Analogue Mood Scale (VAMS) (Stern, 1997) was used to assess the participant's mood. For each of 8 emotions, participants marked a line to indicate the extent to which they felt the emotion. The emotions assessed were afraid, confused, sad, angry, energetic, tired, happy, and tense; a higher score indicates a higher rating for each emotion.

#### CANTAB

The Cambridge Neuropsychological Test Automated Battery (CANTAB) (Sahakian and Owen, 1992) is a computer administered battery of tasks. After a brief practice exercise using the touch screen, nine tests (described below) were administered. Standardized instructions were read out before commencement of each test. The order was the same for all participants and parallel versions were counterbalanced across testing sessions.

*Simple Reaction Time (SRT)* measures participants' reaction time to a known stimulus at a known location. Using their dominant hand, participants pressed a button as quickly as possible after a square was presented on the screen. The analaysed output variable is mean reaction time.

*Paired Associate Learning (PAL)* assesses visual memory and visual learning. During a presentation phase, six boxes randomly opened and closed one by one to reveal either a pattern or nothing. During the test phase one of the patterns previously shown was presented. Participants touched the screen to match the pattern to the box where it was located previously. This was followed until all six patterns were identified. The number of patterns increased with each trial and terminated after ten consecutive fails. Output variables included in the analyses are total errors and stages completed.

*Verbal Recognition Memory (VRM)* assesses both immediate and delayed memory of verbal material under conditions of free recall and forced choice recognition. During a presentation phase, 12 words appeared one by one and participants read each aloud; they were instructed to remember the words. In the first test phase, participants recalled the words without feedback. In the second test phase, half of the words were those presented previously and the remainder were distractors (total *n* = 12). They were presented one at a time and participants touched the screen to indicate whether or not the word was in the original list. The output variable is the number of words correctly identified.

*Big Little Circle (BLC)* tests comprehension, learning, and reversal. It is a training test for the Intra/Extradimensional set shifting task (IED) and participants follow and then reverse a simple rule. Two boxes were presented, each containing a circle. Initially, participants were instructed to touch the box containing the little circle. After a number of trials, they were then instructed to touch the box containing the big circle. The analysed output variable is percentage correct.

*Intra-Extra Dimensional Set Shift (IED)* tests rule acquisition and reversal. Participants must make visual discriminations, maintain attentional sets and have shifting and flexible attention. In this task four boxes were simultaneously presented on the screen, two of which contained a pattern. After touching one of the patterns, participants were given feedback that was used to ascertain the rule that was being used to present the patterns. The rule changes after several correct answers and participants must learn the new rule. The duration of the test varies per person, and the test ends if responses are continually incorrect. The output variables are total trials and total errors.

*Rapid Visual Processing (RVP)* is a test of sustained attention. Single numbers were presented rapidly in a box in the center of the screen. During the practice trials, participants pressed a button when they saw the last digit in a target sequence of numbers (i.e., 3, 5, 7). In the test phase, participants had to respond to three target sequences in the same manner. The test lasted for 4 min. The output variable is total correct hits.

*Verbal Recognition Memory 2 (VRM2)* is a forced choice recognition test. This task was presented approximately 20 min after the first VRM test with the same procedure as that used in the second test phase, but with a different set of twelve distracter words. The variable analysed is total correct.

*Choice Reaction Time (CRT)* is similar to SRT, but with the addition of stimulus and response uncertainty, brought about by two possible stimuli and two possible responses. It assesses motor speed and general alertness. In this task an arrow appears on the screen pointing to the left or the right. Participants had to rapidly press a left or right button to indicate which way the arrow was pointing. There are two outcome variables; mean reaction time for items appearing on the right and mean reaction time for those appearing on the left.

### Procedure

Participants took part in the “water” and “no water” condition one week apart. The order of conditions was counterbalanced. In both conditions participants fasted overnight and were instructed to consume no food or drink after 9.00 pm on the evening preceding testing. A pre-screening medical questionnaire was used to identify and exclude any participants for whom this may cause problems, including those with conditions such as kidney problems, diabetes, or pregnancy, or any other condition that would prevent them from doing an overnight fast.

On the day of testing, participants arrived in the morning. After informed consent was taken, all participants were offered a cereal bar to eat (two fruit options, each 117 kcal). When participating in the water condition, they were offered a 500 ml bottle of water to drink. They were encouraged to have a “big drink,” and then the bottle was removed to prevent the participant from having any further water during the testing session. Any remaining water was measured in order to calculate the amount of water drunk. If participants drank the whole 500 ml and asked for more, they were offered a second bottle. When participating in the no water condition, participants were offered nothing.

Participants then filled in the VAMS and thirst scale^1^, and completed the CANTAB tests. At the second testing session, participants took part in the other water condition and tasks were presented in the same order. At the end of the second test session, participants were debriefed and given a £30 gift voucher for their time. CANTAB testing and the mood scales took approximately 1 h to complete.

### Ethics

This study was approved by the University of East London ethics board. Informed consent was obtained from each participant prior to the study commencing.

### Statistical analysis

The primary aim was to investigate the effect of water supplementation on cognitive performance and mood, while taking into account potential effects of the order of water and no water conditions. The omnibus analyses consisted of a series of mixed model Analyses of Variance (ANOVAs) that were conducted for each outcome variable, for which WATER (water, no water) was a within subjects factor, and ORDER (water first, no water first) was a between subjects factor.

Follow up analyses were conducted that investigated whether thirst moderates the effect of water supplementation on cognition. Participants were grouped according to a median split of thirst ratings.

## Results

There were three exclusions; one participant did not complete the testing at both time points and two were administered the same CANTAB form at both time points in error. The final sample size was 34 participants (25 female). In terms of order, equal numbers had the water or no water test first. In terms of CANTAB version, 18 had version A first. There was no confounding: under each CANTAB order, half had the water test first.

All participants except one reported fasting since 9.00 pm the previous evening. The one participant who reported breaking the fast, had a small drink at 9.30 pm, and was not excluded because this was consumed very close to the 9.00 pm deadline. Participants drank a mean of 775.44 ml water (*SD* = 464.00; range 120–2500 ml) with breakfast when they were in the water condition.

### Water consumption and order

The initial analyses employed a mixed model ANOVA of WATER (water/no water) × ORDER (water first/no water first), conducted separately for thirst, mood scales, and each CANTAB test.

#### Thirst and mood scales

Means and SDs for thirst and mood scale ratings are presented in Table 1, along with the results of the omnibus statistical analysis. Participants rated themselves as having greater subjective thirst on the occasion on which they were not offered water; there was no effect of ORDER and no interaction.

**Table 1:** **Thirst and mood scales means, SDs, and F ratios by water condition (water/no water) and order (water first/no water first)**.

**Scale**	**Water first**				**No water first**				**Results from the omnibus statistical analysis; those with *p* < 0.05 in bold**
	**Water**		**No Water**		**Water**		**No Water**		
	***M***	***SD***	***M***	***SD***	***M***	***SD***	***M***	***SD***	
Thirst	6.44	4.21	12.48	3.41	5.07	4.02	11.64	3.68	**Water *F*_(1, 32)_ = 54.95, *p* < 0.001**
									Water × Order, *F*_(1, 32)_ = 0.095, *p* = 0.760
									Order *F*_(1, 32)_ = 1.20, *p* = 0.282
Afraid	0.58	0.72	0.63	0.92	0.88	1.47	1.8	2.29	Water *F*_(1, 32)_ = 3.03, *p* = 0.091
									Water × Order *F*_(1, 32)_ = 2.46, *p* = 0.127
									Order *F*_(1, 32)_ = 2.87, *p* = 0.100
Confused	1.18	1.25	0.68	0.88	1.66	1.66	2.88	2.48	Water *F*_(1, 32)_ = 1.42, *p* = 0.243
									**Water × Order *F*_(1, 32)_ = 8.12, *p* = 0.008**
									**Order *F*_(1, 32)_ = 7.52, *p* = 0.010**
Sad	0.98	1.20	0.81	1.13	1.34	1.41	2.30	2.69	Water *F*_(1, 32)_ = 0.976, *p* = 0.331
									Water × Order *F*_(1, 32)_ = 1.94, *p* = 0.17
									**Order *F*_(1, 32)_ = 4.54, *p* = 0.041**
Angry	0.92	1.18	0.86	1.24	1.62	2.18	1.81	2.01	Water *F*_(1, 32)_ = 0.028, *p* = 0.865
									Water × Order *F*_(1, 32)_ = 0.116, *p* = 0.736
									Order *F*_(1, 32)_ = 3.26, *p* = 0.080
Energetic	4.29	2.88	3.58	3.16	4.22	2.81	4.01	3.43	Water *F*_(1, 32)_ = 0.87, *p* = 0.359
									Water × Order *F*_(1, 32)_ = 0.25, *p* = 0.620
									Order *F*_(1, 32)_ = 0.036, *p* = 0.751
Tired	4.23	3.20	3.38	3.01	3.05	2.64	5.06	3.03	Water *F*_(1, 32)_ = 0.69, *p* = 0.414
									**Water × Order *F*_(1, 32)_ = 4.16, *p* = 0.050**
									Order *F*_(1, 32)_ = 0.103, *p* = 0.751
Happy	7.02	2.02	6.88	2.57	6.43	2.47	6.06	3.24	Water *F*_(1, 32)_ = 0.269, *p* = 0.684
									Water × Order *F*_(1, 32)_ = 0.033, *p* = 0.856
									Order *F*_(1, 32)_ = 1.02, *p* = 0.320
Tense	1.55	1.33	1.09	1.41	2.06	1.81	2.75	2.18	Water *F*_(1, 32)_ = 0.091, *p* = 0.764
									Water × Order *F*_(1, 32)_ = 2.31, *p* = 0.139
									**Order *F*_(1, 32)_ = 6.89, *p* = 0.013**

In the case of the VAMS, most of the responses were not affected by WATER or ORDER. There were effects of ORDER on scales that asked participants to rate whether they were confused, sad, or tense; participants rated themselves as more tense, more sad and more confused when they had the no water first order. In the case of confused, this was modified by whether they had a drink of water; they were less confused if they had a drink. All other effects of mood were not statistically significant.

#### CANTAB

Means and SDs for all CANTAB tests are presented in Table 2, along with the results of the omnibus statistical analysis. The most clear-cut findings were for SRT and Intra/Extra dimensional set shift.

**Table 2:** **CANTAB tests means, SDs, and *F* ratios by water condition (water/no water) and order (water first/no water first)**.

**Task**	**Water first**				**No water first**				**Results from the omnibus statistical analysis; those with *p* < 0.05 in bold**
	**Water**		**No Water**		**Water**		**No Water**		
	***M***	***SD***	***M***	***SD***	***M***	***SD***	***M***	***SD***	
SRT mean RT^1^, ^2^	263.94	53.03	267.58	50.37	242.63	24.80	278.09	61.32	**Water *F*_(1, 32)_ = 6.99, *p* = 0.013**
									**Water × Order *F*_(1, 32)_ = 4.63, *p* = 0.039**
									Order *F*_(1, 32)_ = 0.126, *p* = 0.725
IED stages completed	7.47	2.58	8.53	0.94	8.59	1.70	8.65	1.46	**Water *F*_(1, 32)_ = 5.39, *p* = 0.028**
									**Water × Order *F*_(1, 32)_ = 4.26, *p* = 0.047**
									Order *F*_(1, 32)_ = 1.23, *p* = 0.275
MOT mean error	9.31	2.93	10.65	2.64	11.44	2.67	9.74	2.63	Water *F*_(1, 32)_ = 0.135, *p* = 0.716
									**Water × Order *F*_(1, 32)_ = 10.02, *p* = 0.003**
									Order *F*_(1, 32)_ = 0.598, *p* = 0.441
MOT mean RT	898.99	148.30	745.67	161.91	718.89	147.74	827.13	201.62	Water *F*_(1, 32)_ = 0.58, *p* = 0.452
									**Water × Order *F*_(1, 32)_ = 19.49, *p* < 0.001**
									Order *F*_(1, 32)_ = 1.02, *p* = 0.319
PAL total errors	3.24	4.66	1.88	3.43	1.41	1.73	3.06	3.05	Water *F*_(1, 32)_ = 0.094, *p* = 0.761
									**Water × Order *F*_(1, 32)_ = 9.80, *p* = 0.004**
									Order *F*_(1, 32)_ = 0.094, *p* = 0.761
PAL stages completed	5.00	0.00	4.94	0.24	5.00	0.00	5.00	0.00	Water *F*_(1, 32)_ = 1.00, *p* = 0.325
									Water × Order *F*_(1, 32)_ = 1.00, *p* = 0.325
									Order *F*_(1, 32)_ = 1.00, *p* = 0.325
VRM recall total correct	9.12	1.80	8.42	2.15	8.88	1.62	9.00	1.73	Water *F*_(1, 32)_ = 0.74, *p* = 0.396
									Water × Order *F*_(1, 32)_ = 1.45, *p* = 0.237
									Order *F*_(1, 32)_ = 0.11, *p* = 0.741
BLC % correct	100	0.00	100	0.00	99.71	0.83	100	0.00	Water *F*_(1, 32)_ = 2.13, *p* = 0.154
									Water × Order *F*_(1, 32)_ = 2.13, *p* = 0.154
									Order *F*_(1, 32)_ = 2.13, *p* = 0.154
RVP total hits	19.29	4.69	20.82	4.43	22.24	4.02	19.24	4.56	Water *F*_(1, 32)_ = 1.23, *p* = 0.276
									**Water × Order *F*_(1, 32)_ = 11.67, *p* = 0.002**
									Order *F*_(1, 32)_ = 0.244, *p* = 0.624
CRT right mean RT	327.62	69.66	318.42	51.02	313.18	43.01	324.77	46.23	Water *F*_(1, 32)_ = 0.021, *p* = 0.886
									Water × Order *F*_(1, 32)_ = 1.57, *p* = 0.220
									Order *F*_(1, 32)_ = 0.061, *p* = 0.806
CRT left mean RT	351.71	86.74	333.06	65.66	315.28	50.34	328.72	44.63	Water *F*_(1, 32)_ = 0.073, *p* = 0.789
									Water × Order *F*_(1, 32)_ = 2.77, *p* = 0.106
									Order *F*_(1, 32)_ = 1.07, *p* = 0.309
PAL 8	8.18	11.64	7.06	9.44	4.29	4.48	4.77	2.88	Water *F*_(1, 32)_ = 0.061, *p* = 0.807
									Water × Order *F*_(1, 32)_ = 0.365, *p* = 0.550
									Order *F*_(1, 32)_ = 1.67, *p* = 0.205
VRM recall total novel	0.18	0.39	0.35	0.61	0.18	0.39	0.24	0.44	Water *F*_(1, 32)_ = 1.12, *p* = 0.297
									Water × Order *F*_(1, 32)_ = 0.281, *p* = 0.600
									Order *F*_(1, 32)_ = 0.262, *p* = 0.612
VRM 2 recognition immediate correct	11.82	0.39	11.59	0.62	11.29	1.05	11.71	0.69	Water *F*_(1, 32)_ = 0.272, *p* = 0.606
									Water × Order *F*_(1, 32)_ = 3.65, *p* = 0.065
									Order *F*_(1, 32)_ = 1.28, *p* = 0.27

1 The analysis of SRT median latency showed the same significant main effect and interaction as that observed for mean SRT.

2 All reaction times are in milliseconds.

On SRT there was a significant main effect of WATER and a significant interaction with ORDER. Participants had faster mean reaction times on the occasion on which they drank water, compared to the occasion on which they did not have a drink of water. The WATER × ORDER interaction was consistent with a practice effect. The water effect was only clearly observable in the condition where water was last. Follow up *t*-tests confirmed these impressions. Reaction times were shorter in the water condition compared to the no water condition, but only if the water condition came second, *t*_(16)_ = 2.71, *p* = 0.016; there was no difference between water and no water groups on the occasion on which they had the water condition first, *t*_(16)_ = 0.53, *p* = 0.603.

In the case of Intra/Extra dimensional set shift (IED; stages completed) there was also a main effect of WATER and WATER × ORDER interaction. Fewer stages were completed by the participants after they had consumed water, but this was only for those who had the water condition first. Follow up *t*-tests supported these impressions. Significantly fewer stages were completed in the water compared to the no water condition when the water condition came first, *t*_(16)_ = 2.20, *p* = 0.043, but there was no significant group difference when the water condition came second, *t*_(16)_ = 1.0, *p* = 0.332.

Performance on Rapid Visual Information Processing (RVP; total hits and misses) was consistent with the explanation that it was affected by practice such that more hits and fewer misses were made in the condition that went second.

Performance on the Motor Control task shows a significant WATER × ORDER interaction for both errors and latency, suggesting more errors and faster responding on the second test. This suggests a speed-accuracy trade off on the second test.

As can be seen from the data presented in Table 2, the remaining CANTAB tests were not affected by WATER or ORDER. Performance on PAL Stages Completed and Big Circle/Little Circle% correct seem to be at ceiling.

### Did thirst moderate the effect of water supplementation?

Follow up analyses investigated whether thirst moderated the effect of water on cognitive performance. Participants were grouped as thirsty or not thirsty by taking the median split of the thirst scale reported on the no water day. Mixed model ANOVAs were conducted on all CANTAB tests and mood scales, for which WATER (water/no water) was a within subjects factor and THIRST (thirsty/not thirsty) was a between subjects factor.

**Figure 1:**
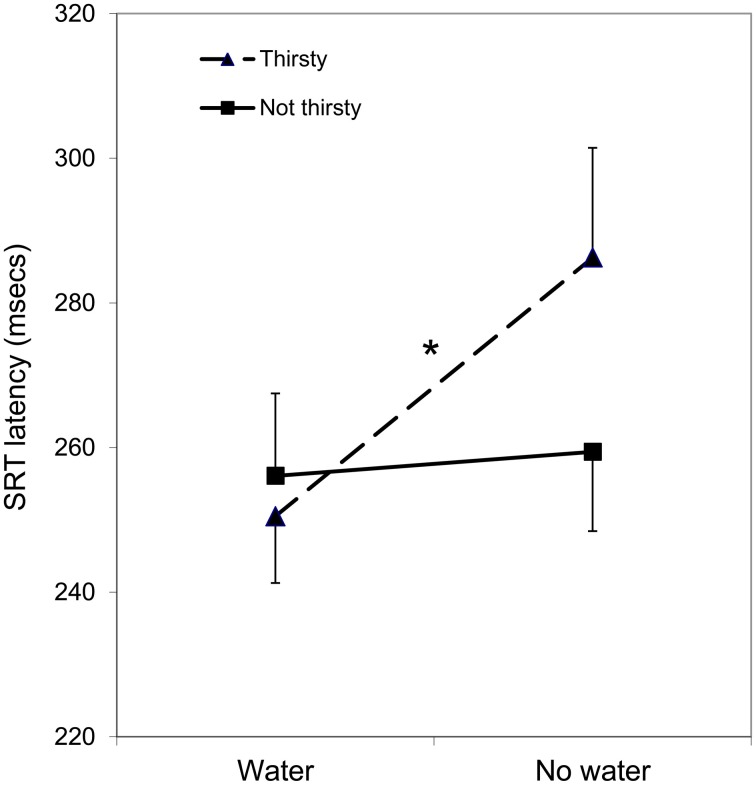
**Mean RT (correct trials) on the SRT task as a function of water condition (water/no water) and thirst (thirsty/not thirsty)**. Asterisk indicates a statistically significant simple main effect of WATER, restricted to the Thirsty group.

There were significant finding for only two CANTAB tests, SRT, and IED. In the case of SRT, there was a significant main effect of WATER, *F*_(1, 32)_ = 7.03, *p* = 0.012, and a significant interaction between WATER and THIRST, *F*_(1, 32)_ = 4.85, *p* = 0.035. The main effect of THIRST was not statistically significant, *F*_(1, 32)_ = 0.494, *p* = 0.487. The significant interaction is illustrated in Figure 1, which indicates that individuals in the low thirst group showed similar response times for both the water and no water testing days. Conversely, those participants who reported being thirsty showed elevated response times (slower) on the no water day, *t*_(16)_ = 2.61, *p* = 0.019. Furthermore, on the occasion on which they had water, their response times were as fast as the non-thirsty group, *t*_(16)_ = 0.61, *p* = 0.551.

**Figure 2:**
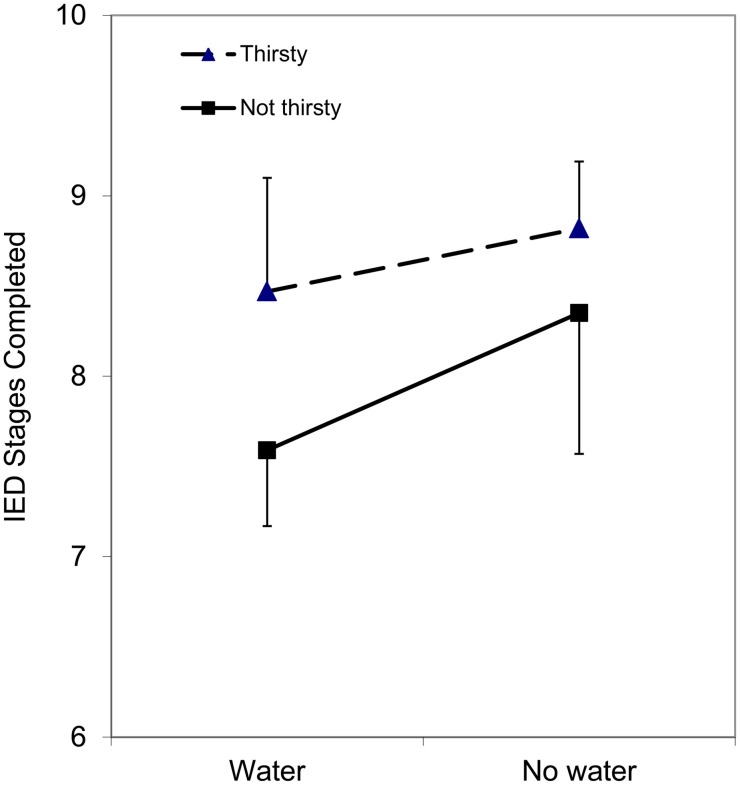
**Mean RT (correct trials) on the Intra-Extra Dimensional Set Shift task as a function of water condition (water/no water) and thirst (thirsty/not thirsty)**. Note that the main effect of WATER was statistically significant, and was not moderated by THIRST.

In the case of IED, there was a significant main effect WATER, *F*_(1, 32)_ = 4.79, *p* = 0.036, with performance better on the no water day than the water testing day. This main effect is illustrated in Figure 2. The main effect of WATER did not interact with thirst, *F*_(1, 32)_ = 0.65, *p* = 0.426, suggesting that subjective thirst is not moderating this effect. The main effect of THIRST was not significant, *F*_(1, 32)_ = 1.49, *p* = 0.231.

In the case of the mood ratings, ratings of “tiredness” and “tense” were higher if individuals were thirsty compared to those who were less thirsty [Tiredness, *F*_(1, 30)_ = 5.82, *p* = 0.022; Tense, *F*_(1, 30)_ = 6.23, *p* = 0.01]. In neither case was there a significant interaction between THIRST and WATER. However, ratings of happiness did show a significant interaction between THIRST and WATER, *F*_(1, 30)_ = 4.62, *p* = 0.040. This was a rather counterintuitive finding in which those who were less thirsty had higher happiness ratings after having water (*M* = 7.1) compared to no water (*M* = 6.2), and those who were more thirsty had higher happiness ratings after no water (*M* = 7.3) compared to having water (*M* = 5.9); however, the follow up *t*-tests were not statistically significant, so not too much weight should be placed on these findings.

## Discussion

The results of the present study show that water supplementation has a positive effect on performance on a SRT task, and that this is moderated by participants' subjective feelings of thirst. Participants who were not thirsty showed a similar speed of responding whether or not they had water to drink. In contrast, participants who rated themselves as thirsty performed at a similar level to non-thirsty participants after a drink of water, but were slower if they did not have a drink. Thus, in thirsty individuals, having a drink of water seems to bring reaction times to a level commensurate with those of non-thirsty individuals, rather than the water making them respond even more quickly. This is strikingly different from the results from the IED task, which showed that performance on the set shifting task was poorer after having water, compared to the occasion on which they did not have water, and that this was not moderated by subjective ratings of thirst.

Thirst moderates the effect of water on some aspects of cognitive performance. Cohen (1983) proposed that dehydration negatively affects cognitive performance, because thirst detracts attention from performance. This explanation is based on a capacity model of information processing, such as that described by Kahneman's (1973) model of attention, which suggests that attention is a finite resource and processing capacity used by one process will result in less being available for others. Thus, the findings of the present study imply that, for speed of responding at least, the positive effects of fluid supplementation may result from an attenuation of the central processing resources that are consumed by the subjective sensation of thirst that otherwise impair the execution of speeded cognitive processes.

While speeded processes were improved by water supplementation, particularly in the case of thirsty individuals, for the controlled processes required by performance on the IED task, performance was facilitated by thirst. Similarly, previous research has found that performance on some cognitive tests appears to be impeded by water supplementation. For example, Edmonds et al. (2013) found that backwards digit span improved from baseline in those offered no water, while showing a very small change in those supplemented with water. D'Anci et al. (2006) suggested that the positive and negative effects of water supplementation on cognitive performance may be explained by the underlying physiological processes, which can have excitatory or inhibitory effects. For example, vasopressin activates the thirst response and has been linked to attention and arousal (Van Londen et al., 1998). Thus, for some aspects of performance, thirst may lead to better performance.

Participants rated themselves as more tired and tense if they were thirsty, but there were minimal effects of thirst or water supplementation on the mood measures used in the present study. This may be because water supplementation and thirst have very little effect on subjective mood. This is in line with studies reported previously; while links have been reported between dehydration and self-rated mood (Shirrefs et al., 2004), previous studies on water supplementation have not found that supplementing with water affects mood (Edmonds et al., 2013). Alternatively, it could be that the measure chosen to rate mood was not sufficiently sensitive and this could be explored in future studies.

Our results showed significant interactions between water supplementation and order that were indicative of both water and practice influencing performance. Taking SRT performance as an example, participants that experienced the water condition second showed faster response times under the “water” condition than the “no water” condition. Whereas, participants that experienced the “water” condition first showed no significant difference between conditions, although the “water” group was slightly faster; this is likely to be because performance in the no water condition benefited from practice. Thus, these results suggest that both practice *and* water consumption played a role, with practice counteracting the effect of water consumption for the water first group. In order to avoid practice influencing results, future studies could adopt the baseline-test design used in other studies (Edmonds and Burford, 2009), and manipulate water supplementation as a between subjects variable.

In the studies conducted on water supplementation and cognition in both adults and children, there are few constants in the research design. This variation in study design is both problematic and prudent. It can be difficult to make comparisons across studies when variables such as the amount of water offered the interval between water supplementation and test, the age of the sample, and the cognitive tests used, all vary. However, in a developing research area, in order to avoid missing effects, it is important not to restrict the study design too soon. This is particularly important when considering the areas of cognition assessed. While there are many differences, there are some constancies that should be incorporated into further studies. For example, letter cancellation, as a test of visual attention and processing speed, has been used in many studies (Edmonds and Burford, 2009; Edmonds and Jeffes, 2009; Booth et al., 2012; Edmonds et al., 2013), and performance on this task reliably shows an improvement at 20, 30, and 40 min post supplementation. Future studies should seek to evaluate the study parameters outlined above in a systematic way.

In conclusion, the present study revealed water consumption to have contrasting effects on different cognitive processes. Water consumption was found both to impair set shifting performance, and to facilitate speed of responding, but in a manner that was dependent upon subjective thirst. More specifically, water consumption appeared to have a corrective effect on the response times for thirsty individuals, bringing their speed of responding up to the level of non-thirsty individuals. This moderating effect of subjective thirst occurred despite participants being asked to abstain from consuming fluids overnight, with the aim of ensuring that all participants arrived at the laboratory with a degree of mild voluntary dehydration. These results are consistent with the facilitative effects of water consumption arising from the freeing up of attentional resources that were otherwise occupied with processing the sensations of thirst. Practice effects also influenced performance, but there was an effect of water supplementation over and above the effects of practice. Further work should examine how this is mediated by thirst mechanisms, as well as determining why water consumption can have negative as well as positive effects on cognitive performance.

### Conflict of interest statement

The authors declare that the research was conducted in the absence of any commercial or financial relationships that could be construed as a potential conflict of interest.

## Footnotes

^1^Bioelectrical impedance was recorded at this point, with the aim of assessing hydration status. Unfortunately, these recordings were not reliable, and will not be reported further.
